# Bioactive Silver Nanoparticles Synthesized Using Endophytic *Bacillus subtilis* CG1 and Their Antimicrobial and Antibiofilm Potential Against Drug-Resistant Pathogens

**DOI:** 10.3390/ph19071102

**Published:** 2026-07-17

**Authors:** Ghaida Saud Aljohani, Saleh H. Salmen

**Affiliations:** Department of Botany and Microbiology, College of Science, King Saud University, P.O. Box 2455, Riyadh 11451, Saudi Arabia

**Keywords:** biosynthesis, bioactive, green nanoparticles, MDR, *B. subtilis* CG1, antimicrobial activity, antibacterial, anticandidal, antibiofilm

## Abstract

**Background/Objectives:** The study addresses the global health challenge posed by multidrug-resistant (MDR) pathogens, highlighting the urgent need for alternative antimicrobial solutions. This study investigated the *in vitro* antimicrobial and antibiofilm potential of endophytic mediated-synthesized silver nanoparticles (AgNPs). **Methods:** An endophytic bacterium was isolated from the medicinal plant *Commiphora gileadensis* in Saudi Arabia and identified as *Bacillus subtilis* CG1 through 16S rRNA gene sequencing. The bacterium was utilized for the green synthesis of AgNPs, as confirmed by Ultraviolet-visible (UV–Vis) spectroscopy. AgNPs characterization was done using Fourier-transform infrared (FTIR) spectroscopy, Transmission and scanning electron microscopy (TEM and SEM), energy-dispersive X-ray spectroscopy (EDX), and dynamic light scattering (DLS). The antimicrobial efficacy of the fabricated AgNPs was tested against eight clinically relevant pathogens using standard *in vitro* assays such as the agar disk diffusion method, minimum inhibitory concentration (MIC), minimum bactericidal and fungicidal concentrations (MBC and MFC). Additionally, AgNPs were tested for antibiofilm activity against *P. aeruginosa* and *S. epidermidis*. Tested pathogens included Methicillin-Resistant *Staphylococcus aureus* (MRSA), *Staphylococcus epidermidis*, *Pseudomonas aeruginosa*, *Klebsiella pneumoniae*, *Escherichia coli*, *Candida auris*, *Candida albicans*, and *Candida tropicalis*. The antibiofilm efficacy was tested using the Crystal violet assay. **Results:** UV–Vis spectroscopy confirmed AgNP formation with a characteristic absorption peak at 412 nm. FTIR analysis identified the presence of hydroxyl, nitrile, and alkyne functional groups, which are involved in nanoparticle reduction and stabilization. TEM and SEM revealed predominantly spherical AgNPs with sizes ranging from 17 to 72 nm, while EDX confirmed silver as the major elemental component. DLS analysis showed a Z-average particle size of 113.9 ± 67.75 nm and a zeta potential of −24.2 mV. The synthesized AgNPs exhibited concentration-dependent antimicrobial activity, producing inhibition zones of 10–20 mm at 240 µg/mL. MIC values ranged from 6.25 to 25 µg/mL, whereas MBC and MFC values ranged from 6.25 to 50 µg/mL and 25 to 100 µg/mL, respectively. Moreover, bacterial growth kinetics analysis demonstrated a concentration-dependent inhibition of growth by AgNPs at MIC and sub-MIC concentrations. Additionally, AgNPs demonstrated significant antibiofilm activity against *P. aeruginosa* and *S. epidermidis.* **Conclusions**: Overall, *B. subtilis* CG1-mediated AgNPs exhibited promising physicochemical properties and antimicrobial and antibiofilm activities, suggesting their potential as alternatives for combating resistant and biofilm-associated infections.

## 1. Introduction

Antibiotic resistance constitutes a significant challenge for the population. Global resistance is influenced by variables including overpopulation, increased global migration, and widespread antibiotic use [1]. Nowadays, antibiotics are the primary method for addressing microbial infections. Regrettably, the emergence of diseases exhibiting increasing antibiotic resistance has signaled the conclusion of the antibiotic discovery era. The persistent failure to develop or discover new antibiotics and irresponsible prescribing are key risk factors contributing to antibiotic resistance [2]. Since the beginning of the twentieth century, following the discovery of penicillin, researchers have persistently sought to develop novel antibiotics to combat bacterial infections caused by bacteria or candida that have developed resistance to current antibiotics [3]. The persistent development of resistance to antimicrobial drugs results in the emergence of multidrug-resistant (MDR) bacteria. *Staphylococcus aureus*, *Acinetobacter baumannii*, *Pseudomonas aeruginosa*, *Enterococcus faecium*, *Klebsiella pneumoniae*, and *Enterobacter* species comprise the group abbreviated as “ESKAPE,” which are the primary agents of nosocomial infections [4]. In addition, *Candida* spp. are among the significant opportunistic fungal pathogens responsible for healthcare-associated infections, particularly in immunocompromised individuals and those undergoing extended antibiotic treatment [5,6]. These diseases, especially invasive candidiasis and candidemia, are linked to considerable morbidity, mortality, and healthcare expenses [6,7]. Prevalent pathogenic species include *Candida albicans*, *Candida tropicalis*, *Candida parapsilosis*, *Candida glabrata*, and the developing multidrug-resistant *Candida auris* [5]. Recent investigations indicate concerning resistance rates in *C. auris*, highlighting extensive resistance to fluconazole and diminished susceptibility to other antifungal classes [5,6]. Novel antimicrobial drugs are necessary for overcoming the resistance of these microbes. Consequently, researchers have investigated the antimicrobial efficacy of silver nanoparticles (AgNPs) to combat that resistance [8,9,10,11].

“Nanoparticles” denotes aggregates of atoms or molecules that assemble into solid and colloidal particles, nano-capsules, and nano-spheres, each exhibiting distinct shapes and dimensions. The dimensions and shape of nanoparticles influence their physical, chemical, and biological characteristics, with diminutive nanoparticles enhancing surface area [12]. Nanoparticles possess diverse uses in the fields of research and medicine. The biosynthesis and production of nanoparticles via bacterial endophytes have emerged as innovative technologies owing to their multifaceted functionality, potential bioactivity, nonpathogenic characteristics, and significant therapeutic utility [13].

Various techniques have been established for the synthesis of AgNPs, among which green synthesis stands out for its ability to yield environmentally advantageous products while minimizing organic waste generation [14]. Bacteria are regarded as very efficient agents for the environmentally friendly synthesis of nanoparticles, especially silver nanoparticles (AgNPs). They synthesize several biomolecules, including enzymes, proteins, and polysaccharides, which function as reducing agents to promote nanoparticle production [15]. The bacterial synthesis of AgNPs offers a sustainable and environmentally friendly alternative to traditional chemical and physical approaches, as it obviates the necessity for hazardous chemicals and energy-intensive processes, hence promoting eco-friendly nanoparticle production and application [16].

Endophytic bacteria are a category of endosymbiotic microorganisms that reside within their plant hosts, fostering a mutually beneficial connection. They offer a host of advantages without compromising other areas of the plant or inducing negative effects. The majority of bacterial endophytes exert beneficial effects, such as the synthesis of bioproducts, phytohormones, and enzymes with diverse applications. They are considered attractive candidates for biological management as natural antagonists. Employing bioproducts generated by microorganisms, including endophytic bacteria, as an alternate method to reduce reliance on synthetic materials and an effective strategy to address pathogen resistance [13].

*Commiphora gileadensis* (Bisham), naturally found in the western and southwestern parts of Saudi Arabia, is regarded as one of the most important medicinal plants of the Arabian Peninsula. Research on Saudi populations of this plant revealed that its extracts contain high levels of flavonoids, terpenoids, phenolics, and volatile oils, all of which contribute to notable biological properties. Alhazmi et al. [17] found that methanolic extracts of *C. gileadensis* showed potent antibacterial activity against clinically significant pathogens such as *Pseudomonas aeruginosa* and *Staphylococcus aureus*, while also enhancing wound healing. In another Saudi investigation, Al-Abdallat et al. [18] analyzed leaves, seeds, callus tissues, and cell suspension cultures, reporting considerable antimicrobial and cytotoxic activities linked to elevated levels of secondary metabolites.

New opportunities for the development of innovative nanomaterials with a broad spectrum of applications are presented by the synthesis of nanoparticles through endophytic intervention. Some endophytic bacteria have developed a unique defense mechanism to combat metal ion toxicity. This method generates nanoparticles by precipitating metal ions at the nanoscale. Certain endophytic bacteria have demonstrated the ability to survive and flourish in environments with elevated metal ion concentrations. Bacteria present a possible avenue for nanoparticle synthesis due to their exceptional ability to transform metal ions into nanoparticles. Endophyte-mediated nanoparticles may exhibit biological effects including antibacterial, antifungal, antioxidant, cytotoxic, and seed germination properties. Nanoparticles synthesized by endophytic bacteria represent a promising strategy due to their diverse biological activities, encompassing antibacterial effects and antibiofilm properties [13].

In this context, the aim of the current study was to isolate and identify endophytic bacteria that are able to biosynthesize AgNPs and assess their antimicrobial and anti-biofilm activities against several multidrug-resistant pathogens.

## 2. Results

### 2.1. Isolation and Identification of Endophytic Bacteria

#### 2.1.1. Morphological and Biochemical Identification

**Figure 1:**
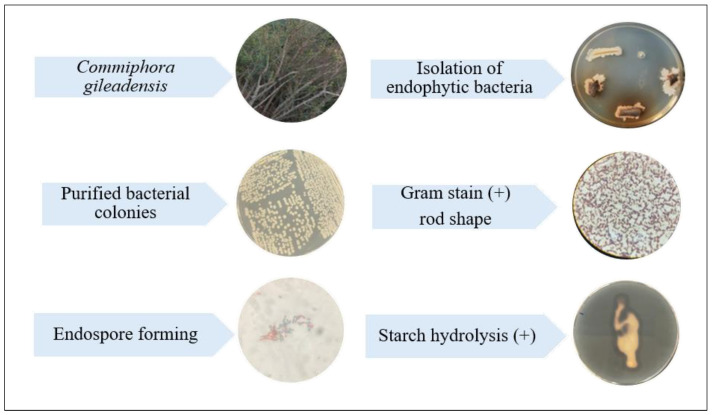
Isolation and phenotypic characteristics of endophytic bacterial colonies from *Commiphora gileadensis*. (+) = Positive.

Endophytic bacteria were isolated from *Commiphora gileadensis* collected from Al Mulaylih, Madinah region, Saudi Arabia (site: 24.8287354, 39.1490872). The bacterial isolate recovered from *C. gileadensis* was characterized using morphological and biochemical analyses. Phenotypic characteristics revealed that the isolate was a Gram-positive, rod-shaped bacterium exhibiting typical *Bacillus*-like morphology (Figure 1). The isolate was catalase-positive and capable of endospore formation. Furthermore, it demonstrated positive starch hydrolysis.

#### 2.1.2. Molecular Identification and Phylogeny of Endophytic Bacteria

**Figure 2:**
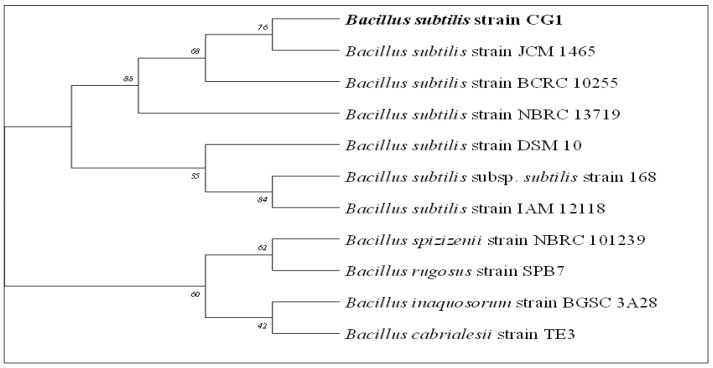
Phylogenetic tree of endophytic bacterial isolate that identified as *Bacillus subtilis* CG1, constructed using 16S rRNA gene sequences.

The endophytic bacterial isolate was successfully identified through molecular identification using 16S rRNA gene sequencing. Sequence analysis demonstrated that the isolate was identified as *Bacillus subtilis* strain CG1 (GenBank accession no.: PZ666008.1). A neighbor-joining phylogenetic tree was constructed utilizing sequencing data from GenBank for strains exhibiting a high percentage of similarity to our strain. (Figure 2).

### 2.2. Green Synthesis of Silver Nanoparticles

**Figure 3:**
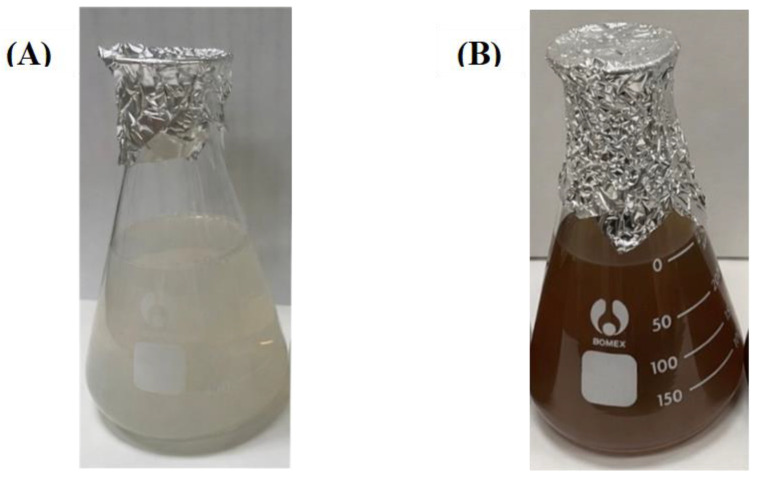
The Successful AgNPs formation indicated by changing the mixture’s color from light yellow (**A**) to dark brown (**B**).

The green synthesis of AgNPs through the reduction of silver nitrate using *Bacillus subtilis* CG1 supernatant was monitored by observing the color change from light yellow to dark brown (Figure 3). The reaction was conducted under temperature 60 °C and pH 10.

### 2.3. Characterization of Silver Nanoparticles

#### 2.3.1. UV Spectral Analysis

**Figure 4:**
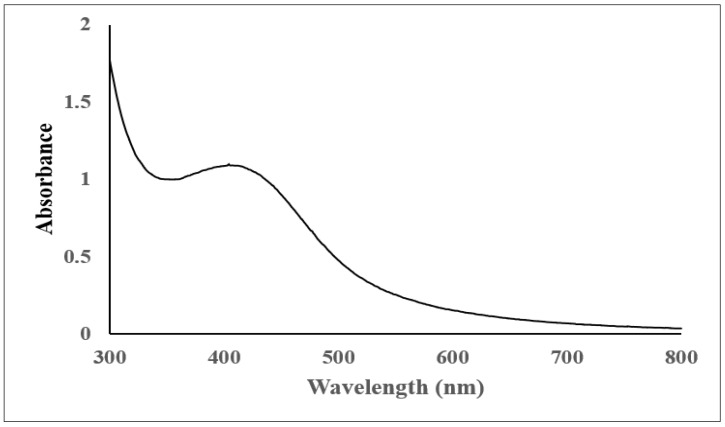
UV-Vis absorption spectrum of *B. subtilis*-AgNPs.

AgNPs synthesis was confirmed using UV–Visible spectrophotometry. The AgNPs exhibited characteristic surface plasmon resonance (SPR) absorption band within the range of 400–420 nm (Figure 4).

#### 2.3.2. FTIR Spectrometer

**Figure 5:**
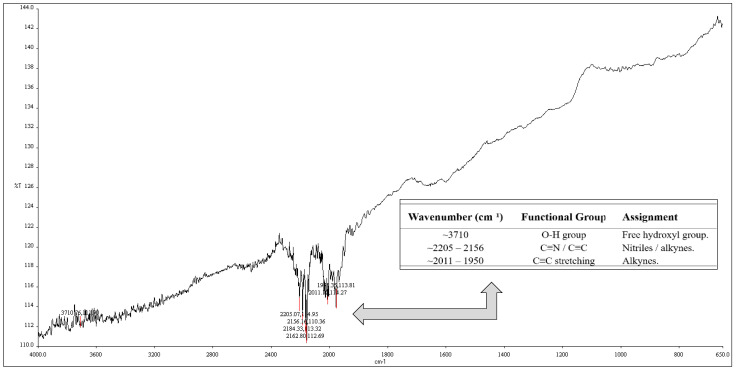
Fourier Transform Infrared (FTIR) spectra of *B. subtilis* CG1-AgNPs recorded in the range of 4000–400 cm^−1^.

The FTIR spectra of *B. subtilis*-AgNPs exhibited several unique absorption bands within the spectrum range of 4000–400 cm^−1^ (Figure 5), verifying the existence of diverse functional groups. An extensive absorption band was detected at around 3710 cm^−1^, signifying O–H stretching vibrations of unbound hydroxyl groups. Additional bands detected in the ranges of 2205–2156 cm^−1^ and 2011–1950 cm^−1^ were assigned to C≡N/C≡C and C≡C stretching vibrations, respectively, signifying the existence of nitrile, alkyne, and associated functional groups.

#### 2.3.3. Transmission Electron Microscope (TEM)

**Figure 6:**
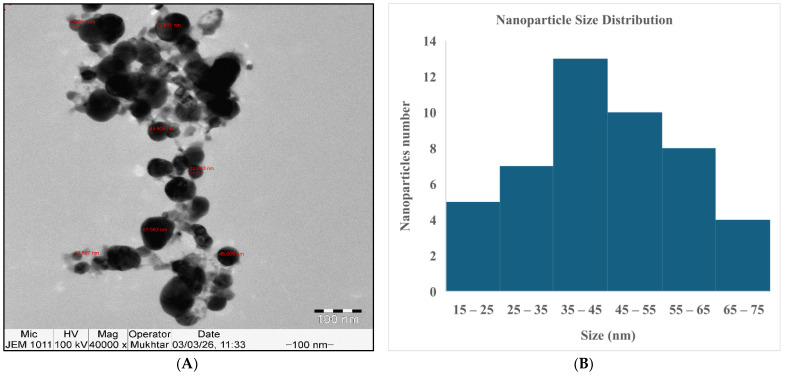
(**A**) Transmission electron micrograph of *B. subtilis* CG1-AgNPs, (**B**) A histogram of the nanoparticle size distribution based on TEM measurements.

Transmission electron microscopy (TEM) analysis confirmed the successful formation of AgNPs in the tested sample. As shown in Figure 6A, the nanoparticles synthesized by *B. subtilis* CG1 appeared spherical with noticeable aggregation forming clusters, and their size ranged from 17.567 to 72.631 nm (Figure 6B).

#### 2.3.4. Scanning Electron Microscopy (SEM) and Energy-Dispersive X-Ray (EDX)

**Figure 7:**
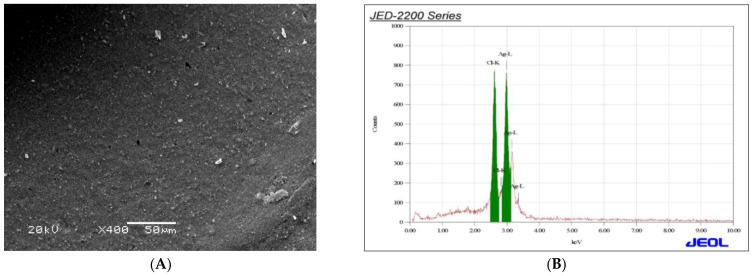
(**A**) Scanning electron microscopy image. (**B**) The elemental composition graph extracted by energy-dispersive X-ray of *B. subtilis*-AgNPs.

The nanoparticles fabricated by *B. subtilis* CG1 were examined using scanning electron microscopy (SEM; JEM-1011) paired with energy-dispersive X-ray (EDX) analysis. SEM imaging was utilized to analyze the surface morphology and structural characteristics of the nanoparticles, as illustrated in Figure 7A. In parallel, EDX analysis provided qualitative and quantitative data regarding the elemental composition of the synthesized materials, verifying the existence of 77.17% silver and 22.83% chloride, which were involved in the synthesis process. Figure 7B presents the spectra displaying separate characteristic peaks for silver (Ag) at 2.983 keV and chloride (Cl) at 2.621 keV. The spectra displayed a strong signal for silver, validating a successful synthesis of AgNPs.

#### 2.3.5. Zeta Size and Potential Determination

**Figure 8:**
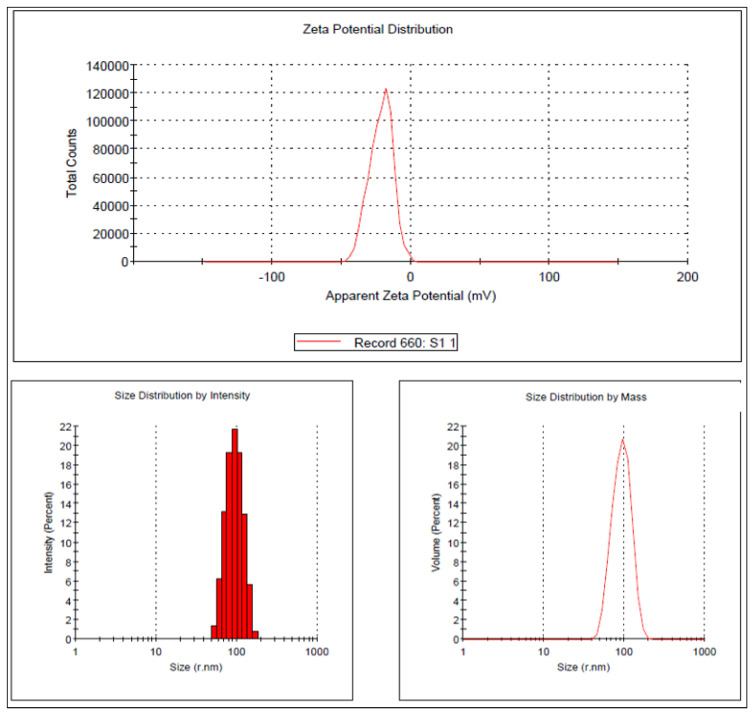
DLS analysis of *B. subtilis* CG1-AgNPs, zeta potential distribution, and size distribution by intensity and mass.

Zeta potential analysis and dynamic light scattering (DLS) were used to examine the surface charge and particle size distribution of the biosynthesized AgNPs produced by *B. subtilis* CG1 (Figure 8). Overall, the synthesized nanoparticles showed negative zeta potential values and a nanoscale size dispersion, suggesting that the bacterial isolate successfully biosynthesized comparatively stable and widely distributed AgNPs. For *B. subtilis*-AgNPs, the zeta potential value was −24.2 mV, while the Z-average particle size was 113.9 ± 67.75 nm. The main peak in the intensity distribution appeared at 95.07 ± 23.93 nm, with a Polydispersity Index (PDI) value of 0.354. These findings confirm that *B. subtilis* CG1 was capable of synthesizing AgNPs with nanoscale dimensions and varying degrees of colloidal stability.

### 2.4. Antimicrobial and Anti-Biofilm Activities of AgNPs

#### 2.4.1. Antibacterial Activity

**Figure 9:**
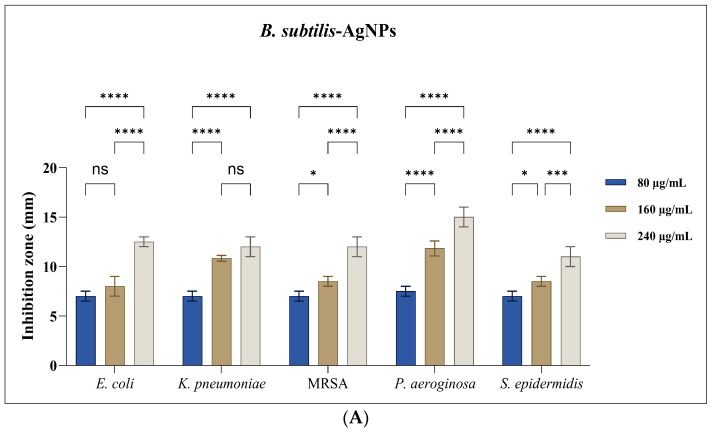

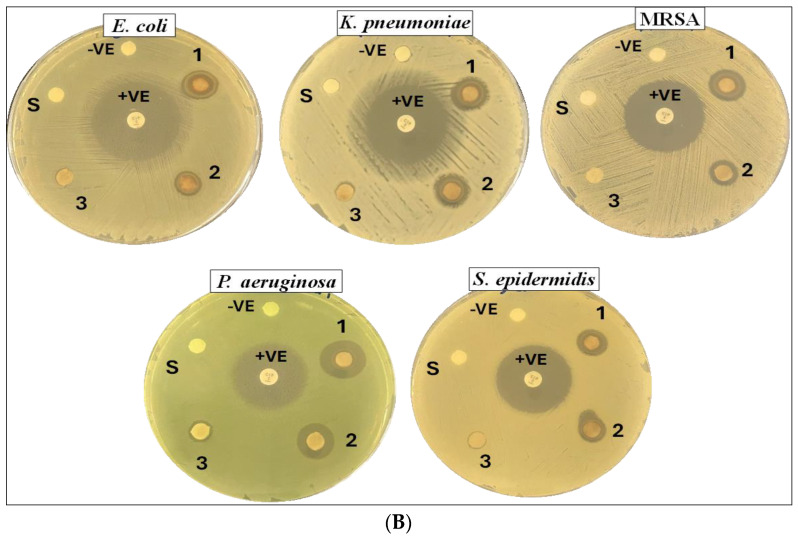
The antibacterial activity of *B. subtilis*-AgNPs against MRSA, *S. epidermidis*, *E. coli*, *K. pneumoniae*, and *P. aeruginosa*. (**A**) Bar chart showing inhibition zone diameters (mm) ± SD (*n* = 3). Statistical significance was assessed using two-way ANOVA (*p* < 0.05) followed by Tukey’s test. Statistical significance is indicated as follows: ns, not significant; * *p* < 0.05; *** *p* < 0.001; **** *p* < 0.0001. (**B**) Representative agar plates demonstrating the inhibition zones. (1) = 240 (µg/mL), (2) = 160 (µg/mL), (3) = 80 (µg/mL), (S) = supernatant, (-VE) = Negative control (0.95% NaCl) and (+VE) = Positive control (Ciprofloxacin).

The antibacterial activity of the biosynthesized AgNPs from *B. subtilis* CG1 exhibited a concentration-dependent inhibitory effect against all tested bacterial strains (Figure 9). The selected AgNPs concentrations (240, 160, and 80 µg/mL) were chosen based on previously reported studies evaluating nanoparticle antibacterial activity against clinically relevant multidrug-resistant bacteria, including methicillin-resistant *Staphylococcus aureus* (MRSA), *Pseudomonas aeruginosa*, *Escherichia coli*, *Klebsiella pneumoniae*, and *Staphylococcus epidermidis*. The highest concentration (240 µg/mL) demonstrated the strongest antibacterial activity, with inhibition zones ranged from 10–16 mm, followed by 8–12 mm at the concentration (160 µg/m), while the lowest concentration (80 µg/mL) exhibited comparatively reduced activity 7–9 mm, its effect was inconsistent across the tested bacterial strains, demonstrating inhibitory activity against some strains while showing no observable effect on others.

#### 2.4.2. Anticandidal Activity

**Figure 10:**
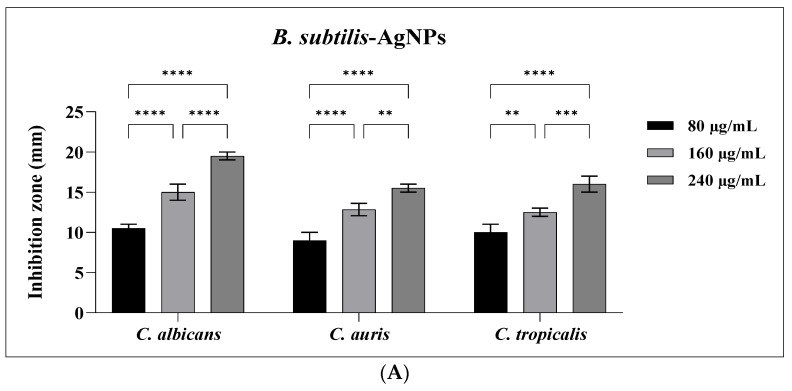

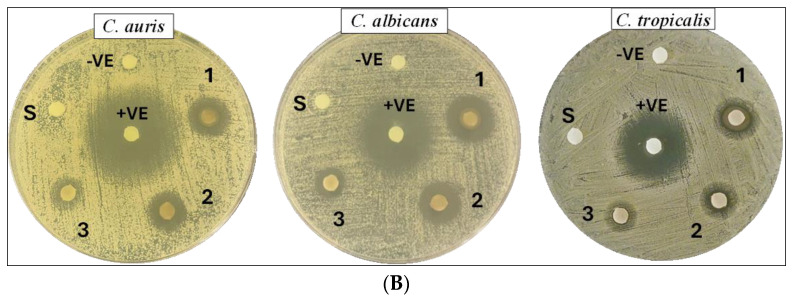
The anticandidal activity of *B. subtilis*-AgNPs against *C. albicans*, *C. auris* and *C. tropicalis*. (**A**) Bar chart showing inhibition zone diameters (mm) ± SD (*n* = 3). Statistical significance was assessed using two-way ANOVA (*p* < 0.05) followed by Tukey’s test. Statistical significance is indicated as follows: ** *p* < 0.01; *** *p* < 0.001; **** *p* < 0.0001. (**B**) Representative agar plate demonstrating the inhibition zones. (1) = 240 (µg/mL), (2) = 160 (µg/mL), (3) = 80 (µg/mL), (S) = supernatant, (-VE) = Negative control (0.95% NaCl) and (+VE) = Positive control (Clotrimazole).

In addition to their antibacterial activity, the fabricated nanoparticles demonstrated inhibitory effects against *C. albicans*, *C. auris*, and *C. tropicalis* (Figure 10), as evidenced by the formation of clear inhibition zones ranging from 15–20 mm at the highest AgNPs concentration (240 µg/mL), followed by 160 µg/mL, with inhibition zones ranging from 12–16 mm, while the lowest concentration (80 µg/mL) showed the smallest inhibition zones, ranging from 8–11 mm. Among the tested candidal species, *C. albicans* showed the highest susceptibility, while *C. auris* was the least. The observed anticandidal activity confirms the ability of the nanoparticles to suppress candidal growth.

#### 2.4.3. MIC, MBC and MFC

The MIC, MBC, and MFC of the biosynthesized AgNPs, derived from *B. subtilis* CG1, were evaluated against the multidrug-resistant bacterial and candidal strains mentioned above. Microbial growth in the MIC assay was assessed visually based on turbidity. The obtained results demonstrated that the antibacterial and anticandidal activity of AgNPs varied depending on the tested microbial strain (Table 1 and Table 2). Overall, MIC values ranged from 6.25 to 25 µg/mL for bacterial strains and 25 µg/mL for all *Candida* species, while MBC and MFC values ranged from 6.25 to 50 µg/mL and from 25 to 100 µg/mL, respectively.

#### 2.4.4. Bacterial Growth Kinetics

**Figure 11:**
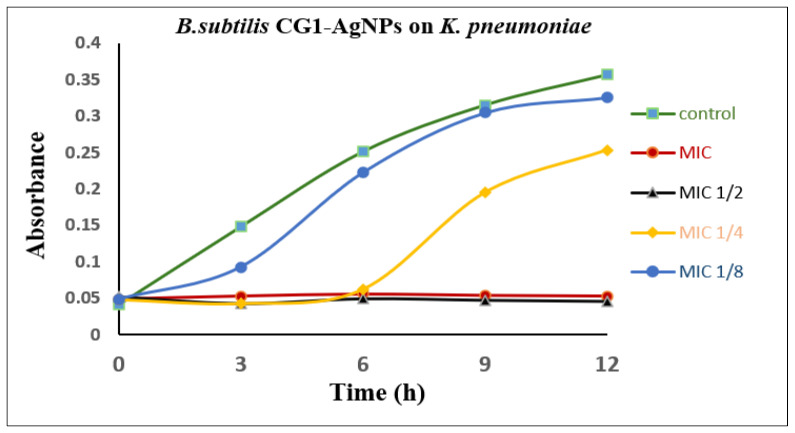
Growth curves of *K. pneumoniae* treated with *B. subtilis* CG1 AgNPs at concentrations of its MIC, 1/2, 1/4, and 1/8 MIC.

**Figure 12:**
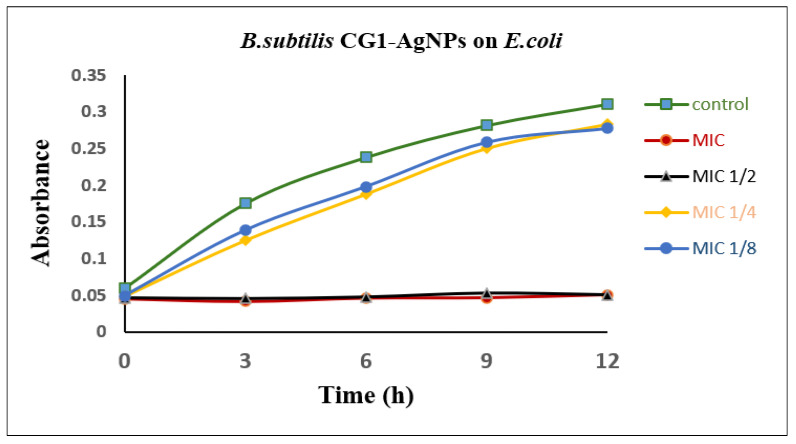
Growth curves of *E. coli* treated with *B. subtilis* CG1 AgNPs at concentrations of its MIC, 1/2, 1/4, and 1/8 MIC.

**Figure 13:**
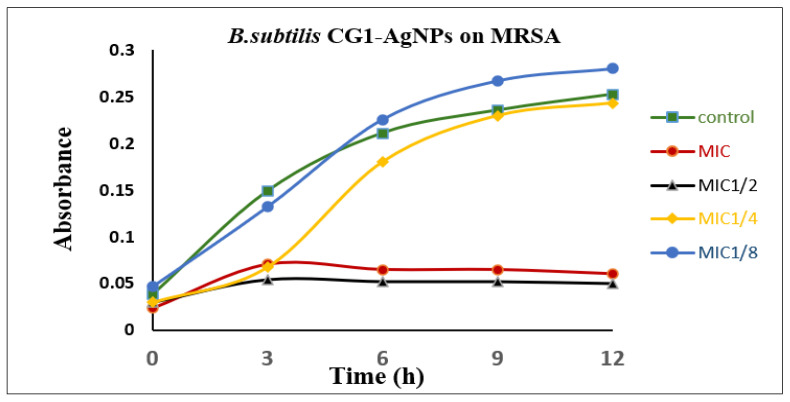
Growth curves of MRSA treated with *B. subtilis* CG1 AgNPs at concentrations of its MIC, 1/2, 1/4, and 1/8 MIC.

**Figure 14:**
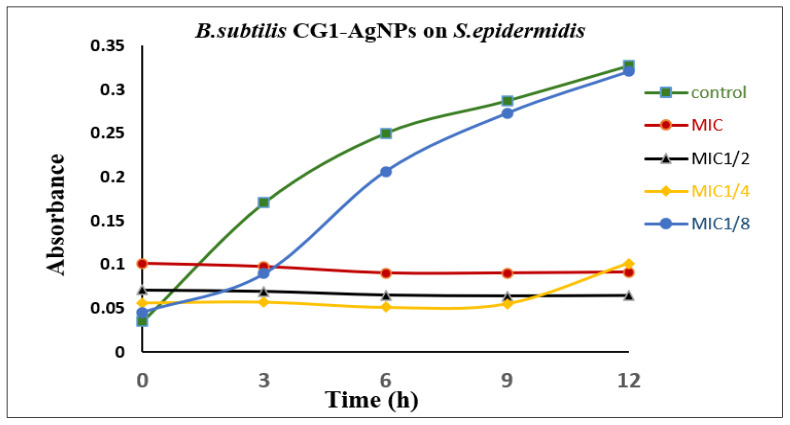
Growth curves of *S. epidermidis* treated with *B. subtilis* CG1 AgNPs at concentrations of its MIC, 1/2, 1/4, and 1/8 MIC.

**Figure 15:**
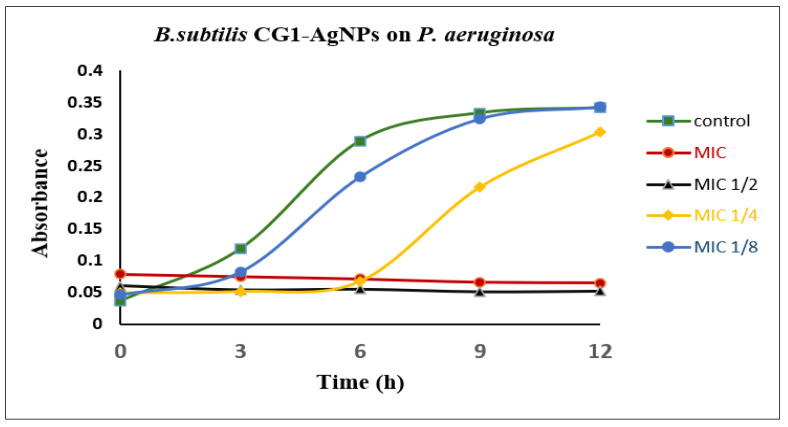
Growth curves of *P. aeruginosa* treated with *B. subtilis* CG1 AgNPs at concentrations of its MIC, 1/2, 1/4, and 1/8 MIC.

Concentrations corresponding to MIC, 1/2 MIC, 1/4 MIC, and 1/8 MIC values were used to assess the impact of *B. subtilis*-AgNPs on bacterial growth kinetics, which were previously determined for each bacterial strain. In general, the growth curves showed that the inhibitory effect of AgNPs against all the bacteria tested was concentration dependent (Figure 11, Figure 12, Figure 13, Figure 14 and Figure 15). The treated cultures with MIC concentrations showed the highest degree of reduction in bacterial growth, either with complete inhibition or with significant suppression of growth throughout the incubation period. Conversely, bacterial cultures treated with sub-MIC concentrations showed different extents of growth retardation by delaying the onset of growth, reducing the growth rates and optical density values compared to the untreated controls. These observations agree with the MIC and MBC results. The gradual recovery of bacterial growth at 1/2 MIC, 1/4 MIC, and 1/8 MIC confirms the concentration-dependent antibacterial activity of AgNPs. The following figures provide detailed growth curves that illustrate the impact of each concentration on the tested bacterial strains.

#### 2.4.5. Antibiofilm Activity

**Figure 16:**
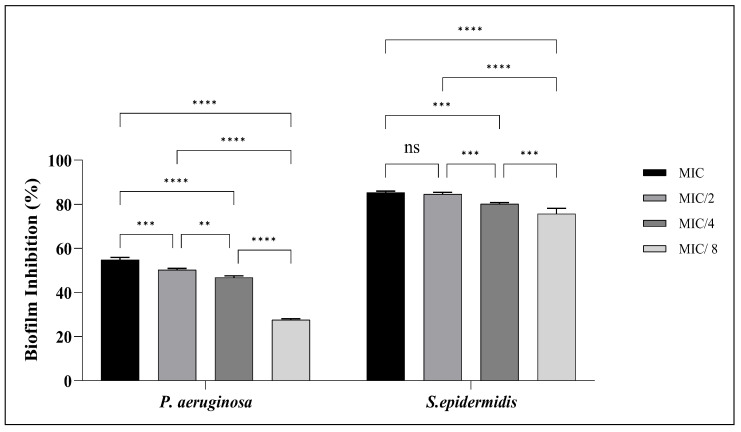
Biofilm inhibition (%) of *P. aeruginosa* and *S. epidermidis* treated with *B. subtilis*-AgNPs at different concentrations. Data are presented as mean ± SD. Statistical analysis was performed using two-way ANOVA (*p* < 0.05). Statistical significance is indicated as follows: ns, not significant; ** *p* < 0.01; *** *p* < 0.001; **** *p* < 0.0001.

**Figure 17:**
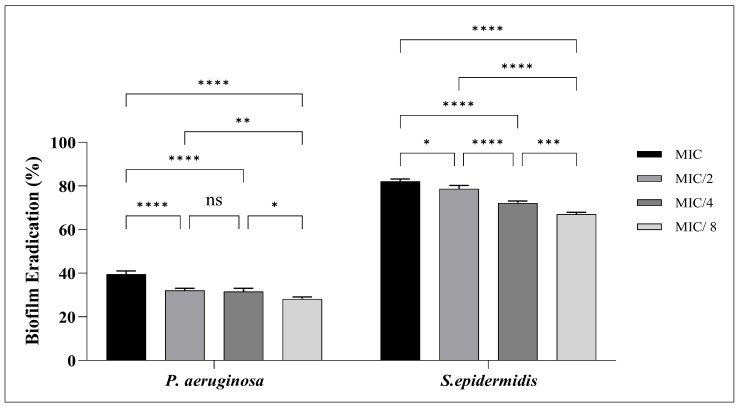
Biofilm eradication (%) of *P. aeruginosa* and *S. epidermidis* treated with *B. subtilis*-AgNPs at different concentrations. Data are presented as mean ± SD. Statistical analysis was performed using two-way ANOVA (*p* < 0.05). Statistical significance is indicated as follows: ns, not significant; * *p* < 0.05; ** *p* < 0.01; *** *p* < 0.001; **** *p* < 0.0001.

The antibiofilm activity of the AgNPs, derived from *B. subtilis* CG1, was evaluated against one Gram-positive bacterium, *Staphylococcus epidermidis*, and one Gram-negative bacterium, *Pseudomonas aeruginosa*, using both inhibition and eradication assays at the same concentrations. The AgNPs demonstrated a concentration-dependent reduction in biofilm biomass, where higher activity was observed at MIC, followed by gradual reduction at lower sub-MIC concentrations among the bacterial strains (Figure 16 and Figure 17).

##### Biofilm Inhibition

AgNPs derived from *B. subtilis* CG1 exhibited significant inhibitory effect on biofilm formation at sub-inhibitory concentrations of MIC, 1/2, 1/4, and 1/8 MIC. For *S. epidermidis*, *B. subtilis*-AgNPs exhibited markedly high inhibition percentages compared to *P. aeruginosa*. At MIC, inhibition value was consistently high, reaching approximately 86%. Even at 1/2 MIC, inhibition remained substantial, approximately 84%, with a gradual decline at 1/4 and 1/8 MIC, yet still maintaining notable activity >70%. In contrast, *P. aeruginosa* showed lower susceptibility. At MIC, inhibition value was approximately 54%. This activity decreased progressively at lower concentrations, reaching approximately 28% at 1/8 MIC.

##### Biofilm Eradication

In contrast to inhibition assay, *B. subtilis*-AgNPs showed reduced efficacy in eradicating preformed biofilms when applied at the same concentrations. For *S. epidermidis*, eradication at MIC reached approximately 83%. Even at 1/2 MIC, eradication remained high, approximately 79%, followed by moderate reductions at 1/4 and 1/8 MIC. Conversely, eradication of *P. aeruginosa* biofilms was notably lower. At MIC, eradication value was approximately 40%, decreasing to 29% at 1/8 MIC.

## 3. Discussion

In response to the growing global challenge of antibiotic resistance, the search for alternative antimicrobial and antibiofilm agents has become increasingly important. In this study, AgNPs were biosynthesized using *B. subtilis* CG1, an endophytic bacterial isolate obtained from *C. gileadensis*, which was collected from Al Mulaylih, Madinah region, Saudi Arabia. The biological activities of *B. subtilis*-AgNPs were evaluated against several pathogenic and multidrug-resistant (MDR) microbial strains. The results demonstrated notable antimicrobial efficacy of the synthesized nanoparticles, highlighting their potential as a promising therapeutic agent and supporting the need for further in vivo investigations.

Endophytic microorganisms are bacteria that inhabit the plants’ internal tissues without causing harm, often forming beneficial associations with their hosts [19]. In the present work, one endophytic bacterial isolate was identified; it was associated with *Bacillus*-related taxa within plant tissues. This finding aligns with recent studies emphasizing the importance of endophytic bacteria as integral components of the plant microbiome, contributing to plant health, growth, and stress resilience [20].

The ecological success of *Bacillus* species as endophytes can be attributed to their distinctive physiological and structural traits. These Gram-positive, spore-forming bacteria are capable of producing highly resistant endospores, enabling survival under adverse environmental conditions such as desiccation, temperature fluctuations, and nutrient scarcity [21]. This resilience likely explains their persistence within plant tissues and their frequent isolation in endophytic studies. Furthermore, a variety of bioactive secondary metabolites, such as lipopeptides, antibiotics, and hydrolytic enzymes, are known to be produced by *Bacillus* species. That molecules contribute to plant growth promotion and the biological control of various pathogens [22,23,24].

In the present study, the isolate was closely related to *Bacillus subtilis* CG1, with sequence similarity values exceeding 99%. This species is widely recognized as a model plant-associated bacterium and has been extensively studied for its plant growth-promoting and biocontrol capabilities [25]. Endophytic bacteria are recognized for producing distinctive secondary metabolites that can serve as effective reducing and capping agents, thereby enhancing nanoparticle stability and enabling controlled size distribution [26]. This likely accounts for the efficient synthesis and stability of the nanoparticles observed in the present study. The findings further demonstrate that AgNP biosynthesis by *B. subtilis* CG1 is strongly influenced by physicochemical parameters, particularly temperature and pH. *B. subtilis*-AgNPs are highly dependent on environmental conditions, with optimal production achieved at 60 °C and pH 10. The results are consistent with earlier reports and emphasize the importance of parameter optimization in enhancing the efficiency, stability, and reproducibility of green nanoparticle synthesis [27,28].

The observed color change was further validated by UV–Vis spectrophotometric analysis. Synthesized nanoparticles displayed a characteristic surface plasmon resonance (SPR) peak within the range of approximately 400–420 nm, confirming the formation of AgNPs. The higher absorbance intensity recorded under optimal conditions reflects an increased nanoparticle concentration, which corresponds with the darker coloration observed visually [29].

The FTIR spectra of the fabricated nanoparticles revealed a broad distribution of functional groups spanning both the high-frequency region (4000–400 cm^−1^) and the fingerprint region (2000–400 cm^−1^), indicating the involvement of diverse biomolecules derived from the bacterial extract. The broad bands observed in the region of ~3800–3500 cm^−1^ are attributed to O–H Free hydroxyl groups, suggesting the presence of hydroxyl-containing compounds. That group is well known to participate in the reduction of metal ions through electron donation mechanisms [30,31]. The presence of these functional groups across the fabricated nanoparticles indicates that the bacterial extract provides a complex biochemical environment facilitating nanoparticle formation via a green synthesis pathway. Overall, the FTIR findings confirm that hydroxyl, nitriles, alkynes, and oxygenated functional groups are primarily responsible for the reduction and stabilization processes. These results are highly consistent with previous studies demonstrating that microbial-mediated nanoparticle synthesis involves synergistic interactions among multiple biomolecular constituents.

Morphological characterization using TEM demonstrated that *B. subtilis*-AgNPs were predominantly spherical, with particle sizes within the nanoscale range from 17–72 nm and exhibited improved dispersion under optimal conditions. Likewise, SEM analysis confirmed the nanoscale structure and surface morphology of the particles, which appeared generally well-dispersed with slight aggregation. The apparent aggregation observed in the TEM images may be attributed to localized particle clustering within the selected imaging field and to the sample preparation and drying process during TEM analysis. In addition, the larger particle size measured by DLS compared with TEM is expected, which DLS measures the hydrodynamic diameter of nanoparticles in suspension, whereas TEM measures the dry particle size [28,31].

Elemental composition analysis using EDX further validated the successful formation of AgNPs. A prominent characteristic peak corresponding to silver (Ag) was detected at about 3 keV, which is indicative of metallic silver [32]. Additionally, signals for chlorine (Cl) were also observed, likely originating from organic biomolecules present in the bacterial extracts that serve as reducing and stabilizing agents during biosynthesis [28,33]. The distinct silver peak, along with these supporting elemental signals, confirms the effective bio-reduction of Ag^+^ ions and the successful synthesis of AgNPs.

Zeta potential analysis further supported the stability of *B. subtilis*-AgNPs. The nanoparticles produced under optimal conditions exhibited a negative zeta potential value, indicating good colloidal stability as a result of electrostatic repulsion between particles [34]. This negative surface charge is commonly attributed to the presence of biomolecules such as polysaccharides and proteins that act as capping and stabilizing agents during biosynthesis [28].

The present study demonstrates that *B. subtilis*-AgNPs exhibit significant antimicrobial activity against multidrug-resistant bacterial and candidal strains, with efficacy strongly influenced by microbial species. Among the tested bacteria, *E. coli* showed the highest susceptibility, with the lowest MIC and MBC values recorded. This enhanced sensitivity may be attributed to the structural characteristics of Gram-negative bacteria, where the outer membrane facilitates nanoparticle interaction and penetration, leading to increased intracellular damage [35]. Previous investigations have revealed similar results that demonstrate strong activity of AgNPs against *E. coli* [36]. In contrast, MRSA and *S. epidermidis* exhibited comparatively higher MIC and MBC values. This may be explained by the thicker peptidoglycan layer in Gram-positive bacteria, which can act as a physical barrier limiting the penetration of nanoparticle. Additionally, resistance mechanisms in these strains may further reduce nanoparticle susceptibility. Notably, *B. subtilis*-AgNPs demonstrated high antibacterial activity, which may be attributed to favorable physicochemical properties enhancing their reactivity and cellular interaction. The close MIC and MBC values observed indicate a bactericidal mode of action, suggesting that the nanoparticles do not merely inhibit bacterial growth but actively induce cell death. This effect is typically linked to various mechanisms, including the loss of cell membrane integrity, the formation of ROS, and interactions with intracellular macromolecules such as proteins and DNA [37].

Alongside the antibacterial efficacy of the produced AgNPs, their antifungal potential was further screened against the three *Candida* species. The nanoparticles exhibited significant anticandidal activity against all the isolates tested, as evidenced by clear zone of inhibition around the treated samples. Responses to nanoparticle exposure were species-dependent as evidenced by differences in susceptibility among the tested species which *C. albicans* showing the highest susceptibility, while *C. auris* was the least. The statistical analysis revealed significant differences between the concentrations of AgNPs tested (*p* < 0.05), indicating that the antifungal effect was dependent on the concentration, with higher concentrations of nanoparticles causing greater inhibition of growth of the *Candida* species tested. These observations suggest that the biosynthesized AgNPs possess a broad-spectrum antimicrobial activity not only against bacterial pathogens but also against fungal microorganisms. Earlier studies have reported similar observations, showing the antifungal activity of AgNPs against clinically important and drug-resistant *Candida* species [38,39]. Multiple modes of action may account for the antifungal efficacy of AgNPs. Silver nanoparticles can compromise the integrity of fungal cell membranes, enhance membrane permeability, and trigger the release of intracellular contents, ultimately leading to cell death [11]. Furthermore, AgNPs induce the production of ROS, resulting in oxidative stress that damages proteins, lipids, and nucleic acids [40]. AgNPs can also interact with biomolecules containing sulfur and phosphorus, disrupting vital metabolic processes and DNA replication, thus inhibiting fungal growth [10]. The activity of AgNPs against resistant *Candida* species further underlines their potential as alternative antifungal agents that can overcome conventional resistance mechanisms.

The bacterial growth kinetics results were in accordance with the MIC and MBC results, confirming the antibacterial efficacy of the synthesized AgNPs. Cultures exposed to concentrations equivalent to the MIC showed a marked suppression of growth, while sub-MIC concentrations resulted in varying degrees of growth delay and decrease in bacterial density. Comparable findings have been documented in earlier studies evaluating the antibacterial activity mediated by nanoparticles. Exposure to nanoparticles delayed the lag phase and reduced the specific growth rate of bacterial cells. The concentration-dependent inhibition observed in the current study indicates the continuous stress of AgNPs on the bacterial populations, thus limiting their ability to multiply and establish normal growth patterns. These results further support the bacteriostatic and bactericidal activities shown by the MIC and MBC assays. Wang et al. [41] also obtained similar results, showing that antimicrobial treatments that produced low MIC and MBC values also significantly altered bacterial growth curves and inhibited bacterial growth. Similarly, El-Habib et al. [42] reported that exposure to nanoparticles resulted in decreased specific growth rates, extended lag phases, and increased antibacterial activity in a concentration-dependent manner.

Biofilm formation is a key determinant in the persistence and resistance of bacterial infections, as biofilm-embedded cells exhibit markedly higher tolerance to antimicrobial agents compared to planktonic counterparts. The complex structure of biofilms, driven by an extracellular polymeric substance (EPS) matrix, acts as a barrier that limits antimicrobial penetration and reduces treatment efficacy. Consequently, targeting both biofilm formation and established biofilms has become an important strategy in controlling biofilm-associated infections. In this context, AgNPs have attracted considerable attention due to their broad-spectrum antimicrobial and antibiofilm properties. In the present study, *B. subtilis*-AgNPs exhibited notable antibiofilm activity against *S. epidermidis* and *P. aeruginosa*. The obtained results demonstrated clear variation in activity depending on the bacterial strain. Both biofilm inhibition and eradication assays showed effective activity, indicating that *B. subtilis*-AgNPs are capable of interacting with different stages of biofilm development. The difference in susceptibility between the tested bacterial strains is consistent with previously reported findings. *S.epidermidis*, as a Gram-positive bacterium, exhibited higher sensitivity compared to *P. aeruginosa*, which is strongly known for its ability to form biofilms and intrinsic resistance mechanisms. The outer membrane structure and dense extracellular matrix of *P. aeruginosa* biofilms contribute significantly to its increased tolerance against antimicrobial agents [43]. The observed antibiofilm activity of *B. subtilis*-AgNPs can be explained by its multiple mechanisms of action. Previous studies have shown that silver nanoparticles can compromise bacterial cell membranes, produce ROS, and obstruct vital cellular functions, in addition to affecting biofilm structure and matrix integrity [44]. Furthermore, AgNPs have been reported to interfere with quorum sensing pathways, thereby reducing bacterial communication and limiting biofilm development [43]. Overall, the present findings agree with earlier studies demonstrating the effectiveness of AgNPs as antibiofilm agents. The ability of the synthesized nanoparticles to act on both developing and established biofilms highlights their potential application in controlling biofilm-associated infections, particularly considering their versatile mechanisms of action and strain-dependent efficacy.

## 4. Materials and Methods

### 4.1. Plant Collection and Identification

Healthy medicinal plant *Commiphora gileadensis* was collected from Al Mulaylih, Madinah region, Saudi Arabia, in February 2026 (site: 24.8287354, 39.1490872). Samples of different parts, such as stems, leaves, and roots, were collected in sterilized closed bags and were transported on the same day to the microbiology research laboratory in the Botany and Microbiology department, King Saud University, for the isolation of endophytic bacteria. Samples of plants were taxonomically authenticated, and a voucher specimen was deposited at the Herbarium of the Department of Botany and Microbiology, King Saud University (KSUH-R.A. Baseni 3159).

### 4.2. Isolation of Pure Culture of Endophytic Bacteria

The plant was gently washed multiple times with sterile distilled water to remove adhering soil and debris. For surface sterilization, the sample was first treated with 70% (*v*/*v*) ethanol for 60 s, followed by immersion in 3% (*w*/*v*) sodium hypochlorite (NaClO) solution for 2 min, and then re-treated with 70% (*v*/*v*) ethanol for an additional 60 s. To eliminate any residual NaClO, the sample was rinsed several times with sterile distilled water (dH_2_O). All sterilization procedures were carried out under aseptic conditions [45].

Sterilized plant segments were then aseptically placed onto Petri dishes containing Nutrient Agar (NA) medium. Each sample was plated in duplicate and incubated at 37 °C for 48 h. Following incubation, emerging bacterial colonies were repeatedly subcultured onto fresh media to obtain pure isolates. The purified bacterial cultures were subsequently preserved at −20 °C in 30% glycerol stocks until further use [46].

### 4.3. Identification of Endophytic Bacterium

#### 4.3.1. Morphological and Biochemical Identification

For morphological characterization, the bacterial isolate was streaked onto Nutrient Agar (NA) plates and incubated at 37 °C for 24 h. Colony characteristics, such as morphology, dimensions, margin, color, elevation, and surface texture, were recorded. A microscopic examination was subsequently performed utilizing freshly cultivated samples [47]. Standard identification protocols were executed in line with Bergey’s Manual of Determinative Bacteriology [48]. Gram staining was performed in accordance with standard methods [49]. Catalase activity was assessed by the conventional hydrogen peroxide assay [47,50]. Starch hydrolysis was assessed by inoculating bacterial colonies onto starch agar plates and incubating them at 37 °C for 24–48 h. Endospore staining was conducted with the Schaeffer–Fulton technique to assess the isolates’ spore production capability [47]. All morphological and biochemical results for isolated bacteria were documented and utilized as initial identification criteria before molecular characterization.

#### 4.3.2. Molecular Identification and Phylogeny of Endophytic Bacteria

Molecular identification was conducted using sequencing of the 16S rRNA gene, a highly conserved genetic marker extensively utilized for bacterial taxonomy and phylogenetic research [51,52]. Fresh cultures preserved in glycerol stock were sent to Macrogen Inc. in Seoul, Republic of Korea, for genetic analysis. The service encompassed genomic DNA extraction and PCR amplification of the 16S rRNA gene with universal bacterial primers; the forward 16S-27F primer (5′-AGAGTTTGATCMTGGCTCAG-3′), and the reverse 16S-1492R primer (5′-TACGGYTACCTTGTTACGACTT-3′); purification of PCR products, and bidirectional sequencing to ensure sequence accuracy and coverage of approximately 1500 base pairs [51]. Raw sequencing reads were assembled into consensus contigs and subjected to similarity analysis. The Basic Local Alignment Search Tool (BLASTn web server) was used to compare the resultant sequences with reference sequences stored in the National Center for Biotechnology Information (NCBI) database [53]. Taxonomic identification was assigned based on the highest percentage similarity, query coverage, and E-value (approaching 0.0). In accordance with commonly accepted thresholds in bacterial taxonomy, a sequence similarity value of ≥98% was considered indicative of close relatedness at the species level, while values below this threshold were interpreted cautiously and reported as closely related species [54,55]. Additionally, Mega 11 program was utilized to perform phylogenetic analysis using the data sequence acquired by Macrogen Inc. Reference sequences from the National Center for Biotechnology Information (NCBI) database, which represent the most closely related species found by Basic Local Alignment Search Tool (BLASTn web server) analysis, were used to build the phylogenetic tree.

### 4.4. Preparation of the Cell-Free Supernatant of Bacillus subtilis CG1

To obtain cell-free supernatant (CFS), *Bacillus subtilis* CG1 was cultured in 250 mL of Nutrient Broth medium and incubated at 37 °C for three days. Following incubation, the culture was centrifuged in a Model J2-21 centrifuge (Beckman Instruments, Palo Alto, CA, USA) at 10,000 rpm for 15 min at 4 °C in order to separate the supernatant. To guarantee total elimination of bacterial cells, the collected supernatant was run through a 0.22 µm membrane filter [56]. 100 µL of the CFS was spread onto Nutrient Agar plates and incubated at 37 °C for 24 h to verify the lack of contamination. The sterile CFS was subsequently stored at 4 °C until further use in the biosynthesis of AgNPs.

### 4.5. Biosynthesis of AgNPs Using Supernatant of Bacillus subtilis CG1

Biosynthesis of AgNP was performed using 50 mL of *Bacillus subtilis* CG1 supernatant that was added to 450 mL of a 3 mM aqueous AgNO_3_ solution in a 750 mL Erlenmeyer flask. Preliminary tests were conducted under different pH and temperature conditions. Based on these preliminary observations, pH 10 and 60 °C were selected as the optimum conditions for AgNP biosynthesis and used in all subsequent experiments. The reaction mixture was adjusted to pH 10 using sterile 1 N NaOH prior to incubation at 60 °C. The flask was incubated in a rotary shaker at 150 rpm for 72 h in darkness to avoid the photoreduction of silver ions. The control sample was created under identical conditions. The treatment was conducted in triplicate. The successful production of nanoparticles was evidenced by a color transition from pale yellow to dark brown. The synthesized AgNPs were recovered using centrifugation at 15,000× *g* for 20 min, purified by washing twice with sterile double-distilled water (ddH_2_O), and subsequently kept at −80 °C for further analysis [57].

### 4.6. Characterization of AgNPs Synthesized Using Bacillus subtilis CG1

The characterization of biosynthesized AgNPs was conducted following the method of Elias et al. [58], with minor modifications, utilizing various techniques. UV–Vis spectroscopy was employed to assess optical density from 200 to 800 nm for evaluation of AgNP synthesis, using a 6705 UV/Vis Spectrophotometer (Jenway, Stone, Staffordshire, UK). The functional groups in AgNPs were determined using FTIR spectroscopy in the 4000–400 cm^−1^ range (Thermo Fisher Scientific, Madison, WI, USA). The morphology and mean dimensions of the AgNPs were assessed utilizing transmission electron microscopy (JEM-1011, JEOL Ltd., Tokyo, Japan). Samples were produced and processed following the manufacturer’s instructions. The chemical composition and elemental composition of AgNPs were measured using a JSM-6380 LA SEM (JEOL Ltd., Tokyo, Japan) and Energy-Dispersive X-Ray (EDX). The size distribution and surface charge of the synthesized AgNPs from the supernatant of *Bacillus subtilis* CG1 were measured using Dynamic Light Scattering (DLS). The average size and surface charge of the AgNPs were determined using a Zetasizer (ZS 90, Malvern Instruments Ltd., Malvern, Worcestershire, UK).

### 4.7. Antimicrobial and Anti-Biofilm Activities of AgNPs

#### 4.7.1. Microbial Cultures

Eight drug-resistant microbial strains obtained from the Microbiology Lab of the Botany and Microbiology Department, College of Science, King Saud University, were used in this study. Microbial cultures included five bacterial strains, which are Methicillin-Resistant *Staphylococcus aureus* (MRSA) (ATCC 43300), *Staphylococcus epidermidis* (ATCC 12228), *Escherichia coli* (ATCC 25922), *Klebsiella pneumoniae* (ATCC 13883), and *Pseudomonas aeruginosa* (ATCC 21584). Additionally, three candidal species were tested, including *Candida albicans* (ATCC 60193), *Candida auris* (ATCC 5130), and *Candida tropicalis* (ATCC 66029). All microbes were sub-cultured on suitable agar plates and incubated at 37 °C for 24–48 h prior to experimentation to ensure viability, purity, and optimal growth conditions.

#### 4.7.2. Preparation of AgNPs Stock Solution

Stock solution of the synthesized AgNPs was prepared by accurately weighing 0.020 g of each dried nanoparticle sample using an analytical balance, followed by dissolution in 5 mL of sterile phosphate-buffered saline (PBS) to obtain a final concentration of 400 µg/mL. The suspension was initially vortexed to ensure preliminary mixing. To achieve uniform dispersion and minimize nanoparticle aggregation, the suspension was subjected to ultrasonic treatment using a probe sonicator (Sonics Vibra-Cell, Sonics & Materials Inc., Newtown, CT, USA) for 10 min under aseptic conditions. Sonication was performed in pulse mode, 5 s ON/5 s OFF, to enhance dispersion efficiency while preventing excessive heating. Following sonication, the AgNPs suspension appeared homogeneous with no visible aggregates, indicating effective dispersion. Following sonication, serial two-fold dilutions were prepared in sterile PBS to obtain a total of ten concentrations suitable for antimicrobial and antibiofilm assays. The dilution series included 400, 200, 100, 50, 25, 12.5, 6.25, 3.125, 1.562, and 0.781 µg/mL, prepared under aseptic conditions to evaluate the concentration-dependent activity of the nanoparticles. AgNPs suspension and dilutions were freshly prepared prior to each experiment to ensure consistency and stability.

#### 4.7.3. Disk Diffusion Method

The antibacterial efficacy of the biosynthesized nanoparticles was assessed via agar disk diffusion method, adhering to conventional protocols with slight modifications [59]. For the antibacterial activity assay, fresh bacterial suspensions were prepared in sterile saline (0.95% NaCl) and calibrated to a 0.5 McFarland standard. Using a sterilized glass bacterial spreader, the suspensions were then evenly spread onto Mueller-Hinton agar plates to create a confluent bacterial lawn. Biosynthesized silver nanoparticles were impregnated onto sterile filter paper disks (6 mm diameter) at concentrations of 240, 160, and 80 µg/mL. Ciprofloxacin 5 µg was used as the positive control, whereas sterile saline (0.95% NaCl) served as the negative control. The cell-free supernatant of *Bacillus subtilis* CG1, employed in nanoparticle synthesis, was independently assessed to determine its intrinsic antibacterial activity compared to the fabricated nanoparticles. All prepared disks were left to dry under aseptic conditions prior to being carefully positioned onto the inoculated agar surface with adequate spacing. The plates were incubated at 37 °C for a duration of 18 to 24 h. Next, a calibrated ruler was used to measure the zones of inhibition surrounding the disks in millimeters.

The synthesized nanoparticles were assessed for anticandidal efficacy against three candidal species using the same methodology. The Mueller-Hinton agar (MHA) medium was enriched with 0.5 µg/mL methylene blue and 2% glucose, subsequently inoculated with standardized candidal solutions, and then treated with nanoparticles at the same concentrations employed for bacterial strains. Clotrimazole at 33 µg/mL was used as the positive control, whereas the negative control was the same as above. All experiments were performed in triplicate to guarantee accuracy.

#### 4.7.4. The Minimum Inhibitory Concentration (MIC), Minimum Bactericidal Concentration (MBC), and Minimum Fungicidal Concentration (MFC)

Following the method outlined by Loo et al. [60] with minimal modifications, the MIC, MBC, and MFC of the biosynthesized AgNPs were assessed in sterile 96-well microtiter plates using the standard broth microdilution technique. Microbial inoculates originated from fresh cultures, standardized to a 0.5 McFarland (~1 × 10^8^ CFU/mL), and subsequently diluted to attain a final working concentration of nearly 1 × 10^6^ CFU/mL. Two-fold serial dilutions of the AgNP were conducted in Mueller–Hinton broth (MHB) to achieve concentrations from 400 to 0.781 µg/mL. To achieve the required final inoculum, equal amounts of the standardized microbial suspensions were thereafter added to each well. Wells with microbial suspension devoid of AgNPs served as growth controls, whilst wells containing purely broth were designated as sterility controls. The plates were incubated at 37 °C for 24 to 48 h. After incubation, microbial growth was assessed visually depending on turbidity; clear wells signified growth inhibition, whereas cloudy wells showed microbial multiplication. The MIC was determined as the lowest concentration of AgNPs that resulted in no observable growth. To determine MBC and MFC, aliquots (10–20 µL) from wells exhibiting no apparent growth were subcultured onto Mueller–Hinton agar plates and incubated under identical conditions. The MBC and MFC were determined to be the minimal concentrations that produced no observable colony formation, signifying total bactericidal and fungicidal efficacy. All experiments were performed in triplicate, and the results were presented as consistent values across independent replicates.

#### 4.7.5. Bacterial Growth Kinetics

In accordance with El-Habib et al. [42], bacterial growth kinetics were evaluated to determine the effect of *B. subtilis*-AgNPs on bacterial proliferation at inhibitory and sub-inhibitory concentrations. Untreated cultures were used as controls, and fresh bacterial suspensions were treated with AgNPs at concentrations corresponding to the MIC, 1/2 MIC, 1/4 MIC, and 1/8 MIC values previously determined for each bacterial strain. Bacterial growth was monitored by measuring the optical density (OD) at 630 nm at 3 h intervals for 12 h. The OD values obtained were used to plot growth curves and to evaluate the change in bacterial growth patterns under treatment with AgNPs.

#### 4.7.6. Anti-Biofilm Activity

##### Biofilm Inhibition Assay

Bacterial suspensions were co-incubated with sub-inhibitory concentrations of the investigated AgNPs at 1 × MIC, 1/2 MIC, 1/4 MIC, and 1/8 MIC, and maintained at 37 °C for 24 h to assess biofilm inhibition. After incubation, the wells were carefully rinsed three times with sterile PBS to eliminate non-adherent (planktonic) cells. The residual adhering biofilm was fixed using methanol, stained with 0.1% (*w*/*v*) crystal violet for 15 min, and subsequently washed with distilled water. The residual stain was subsequently solubilized with 33% (*v*/*v*) acetic acid, and the absorbance was recorded at 630 nm using a microplate reader (MR-96A, Mindray Bio-Medical Electronics Co., Ltd., Shenzhen, China). The biofilm inhibition percentage was calculated relative to the untreated control as follows:

$$\%\;\mathrm{Biofilm}\;\mathrm{inhibition}\;=\frac{O{D\;}_{control}-OD_{\;sample}}{OD_{\;control}}\times 100$$

##### Biofilm Eradication Assay

In the biofilm eradication assay, biofilms were initially produced by incubating bacterial suspensions in Tryptic Soy Broth (TSB) enriched with 1% (*w*/*v*) glucose for 24 h under the same conditions. Subsequent to biofilm growth, the wells were carefully rinsed with PBS to exclude non-adherent cells, followed by the addition of fresh media containing the evaluated AgNPs. Similar nanoparticle concentrations (MIC, 1/2 MIC, 1/4 MIC, and 1/8 MIC) were utilized to facilitate direct comparison of biofilm inhibition and eradication effects. The plates were subsequently incubated for a further 24 h, followed by washing, crystal violet staining, and absorbance measurement as previously reported. The reduction in biofilm biomass was determined relative to untreated biofilm controls as follows:

$$\%\;\mathrm{Biofilm}\;\mathrm{eradication}\;=\frac{O{D\;}_{control}-OD_{\;treated}}{O{D\;}_{control}}\times 100$$

All experiments were conducted in triplicate, and the data were presented as mean ± standard deviation.

### 4.8. Statistical Analysis

Antimicrobial and antibiofilm activity results were obtained in triplicate, and significance (*p* < 0.05) was determined by comparing the means using a two-way ANOVA followed by Tukey’s post hoc test. The data was analyzed using GraphPad Prism 11.0.1 software (GraphPad Software, Inc., Boston, MA, USA).

## 5. Conclusions

This work is the first to describe the simple and environmentally friendly biosynthesis of AgNPs using endophytic *Bacillus subtilis* CG1 isolated from *C. gileadensis* plant in Saudi Arabia. Additionally, it demonstrates the first use of these biosynthesized AgNPs against pathogenic microbes such as MRSA, *S. epidermidis*, *K. pneumoniae*, *E. coli*, *P. aeruginosa*, *C. auris*, *C. albicans*, and *C. tropicalis*.

For the fabrication of silver nanoparticles, the extracellular method, which is straightforward, quick, repeatable, and environmentally friendly was employed under optimal conditions of pH 10 and temperature 60 °C.

Techniques including UV-Vis spectroscopy, TEM, EDX, DLS, and FTIR were used to examine the AgNPs produced by *Bacillus subtilis* CG1. Comprehensive characterization confirmed the successful formation of nanoscale, predominantly spherical AgNPs, stabilized by biologically active functional groups that contribute to their structural integrity and functional performance.

AgNPs synthesis confirmed by UV-visible spectroscopy at a peak of 412 nm. TEM and SEM revealed predominantly spherical nanoparticles, averaging 17 to 72 nm in size, with a uniform distribution. EDX validated silver as a major component, while dynamic light scattering DLS indicated a zeta potential of −24.2 mV and a Z-average particle size of 113.9 ± 67.75 nm, demonstrating good colloidal stability.

Critically, the biosynthesized AgNPs demonstrated potent antibacterial and anticandidal activity against clinically relevant MDR pathogens, exhibiting a clear concentration-dependent effect, with inhibition zones ranging from 10 to 20 mm at the highest concentration (240 µg/mL). MIC values ranged from 6.25 to 25 µg/mL, while MBC and MFC ranged from 6.25 to 50 µg/mL and 25 to 100 µg/mL, respectively.

Moreover, bacterial growth kinetics analysis demonstrated a concentration-dependent inhibition of growth by AgNPs at MIC and sub-MIC concentrations.

Beyond planktonic inhibition, the nanoparticles showed pronounced antibiofilm efficacy, effectively suppressing biofilm formation and disrupting established biofilms, thereby directly targeting a key driver of chronic and treatment-resistant infections.

Collectively, these findings highlight the dual functionality of endophyte-mediated AgNPs as both antimicrobial and antibiofilm agents, positioning them as strong candidates for next-generation antimicrobial strategies. Their effectiveness against MDR pathogens, combined with their green synthesis and physicochemical stability, underscores their translational potential in biomedical and clinical applications.

The antimicrobial and antibiofilm properties of the biosynthesized AgNPs revealed in this investigation are mostly comparable to those previously documented for AgNPs produced with conventional *Bacillus* strains. Comparable antimicrobial efficacy has been documented for AgNPs produced utilizing non-endophytic *Bacillus* isolates, affirming the capability of *Bacillus* species as effective biological nano-factories. Nevertheless, the majority of published investigations have been on previously identified laboratory-derived *Bacillus* strains, while reports of AgNP production utilizing the endophytic *B. subtilis* CG1 remain scarce. This study enhances existing knowledge by showing that a new strain of an endophytic *B. subtilis* isolated from *Commiphora gileadensis* can effectively serve as a biological source for the green synthesis of bioactive AgNPs with notable antibacterial and antibiofilm properties.

While the present findings demonstrate the promising antimicrobial and antibiofilm potential of the biosynthesized AgNPs under in vitro conditions, future studies should focus on evaluating the long-term stability, in vivo efficacy, and biosafety of these AgNPs to further support their potential biomedical applications.

## Figures and Tables

**Table 1:** The MIC µg/mL and MBC µg/mL ± SD of *B. subtilis*-AgNPs against MRSA, *S. epidermidis*, *E. coli*, *K. pneumoniae* and *P. aeruginosa*.

Bacterial Strains	MIC (µg/mL)	MBC (µg/mL)	Growth of Bacterial Colonies
MRSA	25 ± 0.00	25 ± 0.00	** 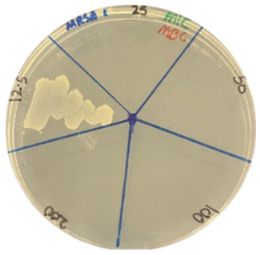 **
*S. epidermidis*	25 ± 0.00	50 ± 0.00	** 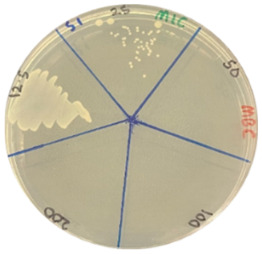 **
*E. coli*	6.25 ± 0.00	6.25 ± 0.00	** 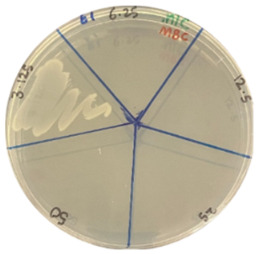 **
*K. pneumoniae*	12.5 ± 0.00	25 ± 0.00	** 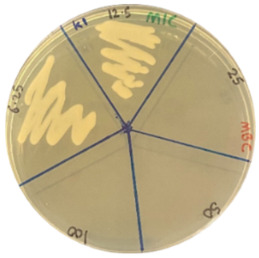 **
*P. aeruginosa*	12.5 ± 0.00	25 ± 0.00	** 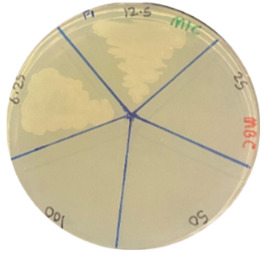 **

**Table 2:** The MIC µg/mL and MFC µg/mL ± SD of *B. subtilis*-AgNPs against *C. albicans*, *C. auris* and *C. tropicalis.*

Candidal Species	MIC (µg/mL)	MFC (µg/mL)	Growth of Candidal Colonies
*C. albicans*	25 ± 0.00	100 ± 0.00	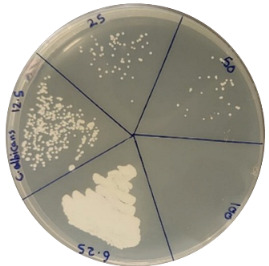
*C. auris*	25 ± 0.00	50 ± 0.00	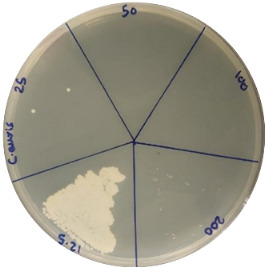
*C. tropicalis*	25 ± 0.00	25 ± 0.00	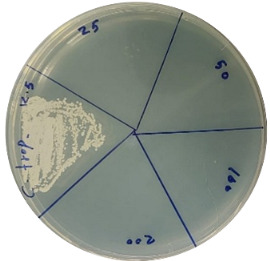

## Data Availability

The raw data supporting the conclusions of this article will be made available by the authors on request.

## Abbreviations

The following abbreviations are used in this manuscript:

AgNPs	Silver Nanoparticles
MDR	Multi-Drug Resistant
MRSA	Methicillin-Resistant *Staphylococcus aureus*
TEM	Transmission Electron Microscopy
SEM	Scanning Electron Microscopy
EDX	Energy-Dispersive X-Ray Spectroscopy
DLS	Dynamic Light Scattering
FTIR	Fourier-Transform Infrared Spectroscopy
AgNO_3_	Silver Nitrate
PBS	Phosphate Buffered Saline
NA	Nutrient agar
MHA	Mueller Hinton Agar
TSB	Tryptone Soy Broth
CFS	Cell-Free Supernatant
MIC	Minimum Inhibitory Concentration
MBC	Minimum Bactericidal Concentration
MFC	Minimum Fungicidal Concentration
